# Quality of life and abdominal wall functionality after abdominal wall reconstruction: A prospective single center follow-up study

**DOI:** 10.1007/s10029-024-03143-4

**Published:** 2024-09-06

**Authors:** M Toma, V Oprea, Florentina Scarlat, Carmen Elena Bucuri, O Andercou, F Mihaileanu, O Grad, M Rosianu, C Molnar

**Affiliations:** 1 Clinical Department of Surgery, “Constantin Papilian” Emergency Clinical Military Hospital, Cluj-Napoca, 22G-ral Traian Mosoiu, Cluj-Napoca, Romania; 2 Pharmacy, Science and Technology, “George Emil Palade” University of Medicine, Targu-Mures, Romania; 3 “Iuliu Hatieganu” University of Medicine and Pharmacy, Cluj-Napoca, Romania; 4 Second Clinical Department of Surgery, Emergency Clinical County Hospital, Cluj- Napoca, Romania; 5 Clinical Department of Radiology - Medical Imaging, Emergency Clinical County Hospital, Sibiu, Romania; 6 First Clinical Department of Surgery, Emergency Clinical County Hospital, Targu- Mures, Romania

**Keywords:** Truncal function, Incisional hernia, EQ-5D score, Rives-Stoppa, ACS, PCS, Quality of life

## Abstract

**Background:**

Fascial breakdown with the occurrence of an incisional hernia (IH) is an important and challenging complication of any laparotomy. For a long time, the success of the abdominal wall reconstruction (AWR) was measured only from the surgeon’s perspective by defining outcome measures such as wound morbidity and recurrence. The understanding that complete recovery is difficult to assess without considering patients has shifted the paradigm of optimal outcomes to Patient Reported Outcome Measures (PROMS) and Quality of Life (QoL), which are pivotal to evaluate the success and efficacy of AWR.

**Methods:**

We conducted a prospective follow-up study of 91 patients undergoing mesh-augmented abdominal wall reconstruction for primary or recurrent incisional hernia between January 2021 and December 2023. Demographic data, comorbidities, and hernia characteristics were recorded. All patients were evaluated preoperatively by a native abdomino-pelvic CT scan to assess the characteristics of hernia (length, width, surface, and volume of the incisional hernia sac and of peritoneal cavity), the presence of mesh (if previously inserted), and abdominal wall muscles status. All intervention were performed by the same surgical team according to the techniques described by Rives – Stoppa (RS), Ramirez (ACS), and Novitsky (PCS). Abdominal wall function was assessed using trunk raising (TR) and double leg lowering (DLL) measurements performed preoperatively, 1 month, 6 months, and 1 year postoperatively. At the same time, pre- and post-operative quality of life was analysed using the EQ-5D score.

**Results:**

Mean age of 59.42 ± 12.28 years and a male/female ratio of 35/56 were recorded, most of them being obese. There were 36 (42%) patients with defects larger than 10 cm. The distribution of the type of surgical intervention was: RS 35 patients, ACS 13 patients, and PCS 43 patients. The mean value of combined score for the preoperative abdominal wall functionality was 4.41 ± 1.67 (2–8) while the mean value of preoperative EQ-5D index was 0.652 ± 0.026 (-0.32–1.00). QoL was poor and very poor for 48% (44) of the patients who recorded index values less than 0.56 (50% percentile). Preoperative EQ-5D index was highly correlated with Combined AWF score (*r* = 0.620; *p* < 0.0001) and the correlation was specific (AUC = 0.799; *p <* 0.0001; asymptotic 95%CI = 0.711–0.923). At 12 months, the AWF score increased to 8.13 ± 2.58 (1–10) and the QoL total score to 0.979 ± 0.007 (0.71–1). Good and very good total scores for QoL were recorded for 47 patients (84%) compared to 33 (36%) in the preoperative evaluation (χ^2^ with Yates continuity correction for two degrees of liberty = 46.04; *p* < 0.00001).

**Conclusion:**

Our results suggest that patients can expect to see a significant overall improvement in all five components of QoL measured with the help of Eq. 5D questionnaire. This improvement is dependent by hernia size, and some individual patient’s factors (diabetes, cardiovascular diseases, and age over 60 years).

**Supplementary Information:**

The online version contains supplementary material available at 10.1007/s10029-024-03143-4.

## Background

Fascial breakdown with the occurrence of an incisional hernia (IH) is an important and challenging complication of any laparotomy. Estimated rates may be as high as 40% in high-risk patients 2 years after midline incision [1, 2].

For a long time, the success of the abdominal wall reconstruction (AWR) was measured only from the surgeon’s perspective by defining outcome measures such as wound morbidity and recurrence [3]. Beyond this, the results of hernia surgery should be complete recovery with a sound, functional abdominal wall [4]. The understanding that complete recovery is difficult to assess without considering patients has shifted the paradigm of optimal outcomes to Patient Reported Outcome Measures (PROMS) and Quality of Life (QoL), which are pivotal to evaluate the success and efficacy of AWR. QoL and recovery of activities of daily living have rapidly become key outcome measures in the field of hernia surgery over the last decade [1, 5].

Assessing QoL in IH repair is challenging, because not all surveys are equally created and may not provide the same amount of information [6]. Many hernia – specific or non-specific instruments have been developed and are available to assess functional health status; a recent review described 14 tools across 32 studies [7]. Six AWH-specific tools were identified through an extensive literature search [8–12] but none of them has been universally accepted and none become dominant [11]. Acceptability (completion rates), reliability (how will a measure will provide similar values for patients with similar QoLs), and validity (how accurately reflects what is supposed to measure) are influential factors that can jeopardize the accuracy of QoL evaluation.

We hypothesize that patients with IH despite the size and location of the defect demonstrate varying degrees of abdominal wall function (AWF) impairment and decreased QoL because of this. Our study aimed to explore how AWF and QoL are improved after different types of AWR. The second goal was to identify which pre – and postoperative factors are independently related and influence QoL.

## Materials and methods

### Patients

We conducted a prospective follow-up study of consecutive patients undergoing abdominal wall mesh augmented reconstruction for primary or incisional hernia between January 2021 and December 2023. The paper’s senior authors (MT and OV) carried out the follow-up examination. All the included patients were over 18 years old with primary or recurrent incisional hernias and agreed to participate after the informed consent and if they completed the preoperative and at least one postoperative quality assessment form. Subsets of umbilical, trocar site, emergent cases, and parastomal hernias were excluded. In addition, patients who refused to participate and those in whom a mesh repair could not be performed (VHWG type 4 – infected mesh patients) were excluded.

Written informed consent was obtained for each patient. The study was carried out under approval Ad 4528–9/25.11.2020 of the Local Ethic Committee. Demographic data (age, gender, ASA classification, Body Mass Index-BMI, comorbidities) and hernia characteristics (previous hernia repair, defect location, dimensions and surface, type of procedure) were recorded from the data files. All patients were evaluated preoperatively by a native abdomino-pelvic computerized tomography (CT) to assess the characteristics of hernia (length, width, surface, and volume of the incisional hernia sac and of peritoneal cavity), the presence of mesh (if previously inserted), and abdominal wall muscles status (width of the rectus muscle for retro-rectal space evaluation, the width and length of the lateral muscles). The full investigational protocol has been reported elsewhere [13]. CT scanning was repeated in each patient after Dysport and pneumoperitoneum administration to assess the effectiveness of procedures.

### Surgical procedure

All patients had intravenous and antithrombotic prophylaxis according to anesthesiologist protocol. Preoperatively, when Sabbagh index was larger than 0.25, the abdominal wall was optimized with the aid of chemical components separation with Botulin Toxin A (Dysport™ Ipsen Pharma 500 IU) injection according to Ibarra – Hurtado technique [14]. Dysport was administered 4 to 6 weeks preoperatively in order to obtain the maximal effect. When larger defects were associated with irreducibility of the hernia, Progressive Preoperative Pneumoperitoneum (PPP) was performed according to a previously described technique [15]. Duration and volume of insufflated air where variable in accordance with local conditions.

All surgical repairs were performed under general anesthesia by the same surgeons (OV and MT) according to the techniques described by Rives - Stoppa [16], Ramirez [17], and Novitsky [18]. The indication for one of the procedures was consistent with the anatomy of the defect and the anatomy of the abdominal wall. After adhesiolysis, the peritoneal sac was dissected, preserved until the final evaluation of the possibility of fascial closure, and the abdominal wall reconstructed. Whenever encountered, the previous mesh was completely removed when possible.

The abdominal wall was reinforced with a medium weight macroporous monofilament polypropylene placed as sublay or as an on lay. The mesh surface varied between 3-to-5-fold defect area respecting the principles of Mesh Defect Area Ratio (MDAR) [19, 20]. The mesh was held in place with synthetic glue fixation (Histoacryl™ B Braun). All patients were drained with two suction drains on the top of the mesh until the volume of the liquid was less than 30 ml for two consecutive days. Excess skin was removed after anterior fascia closure without routine abdominoplasty. Early mobilization was encouraged postoperatively, and discharge was performed after adequate oral intake and pain control with oral analgesics. The drains were removed when the drainage was less than 30 ml for two consecutive days (usually after discharge if the recovery was uneventful).

### Abdominal wall functional (AWF) evaluation

The function of the reconstructed linea alba was evaluated with the aid of previously reported trunk raising (TR) and double leg lowering (DLL) [21]. The test has proven its reliability and we decide to continue using it. A team of two examiners performed all measurements (OV and MT), each performing both assessments. The combined scores (composite or total scores) represent the AWF value and are summarized in Table 1. The AWF measurement was done preoperatively, at 1 month, 6 months, and one –year postoperatively.

**Table 1:** Abdominal Wall Functionality Combined score evaluation (the combined score is the sum of the value of trunk raising (TR) and double leg lowering (DLL)

Trace	1–2
Poor	3–4
Fair	5–6
Good	7–8
Normal	9–10

### Quality of life (QoL) evaluation

**Fig. 1:**
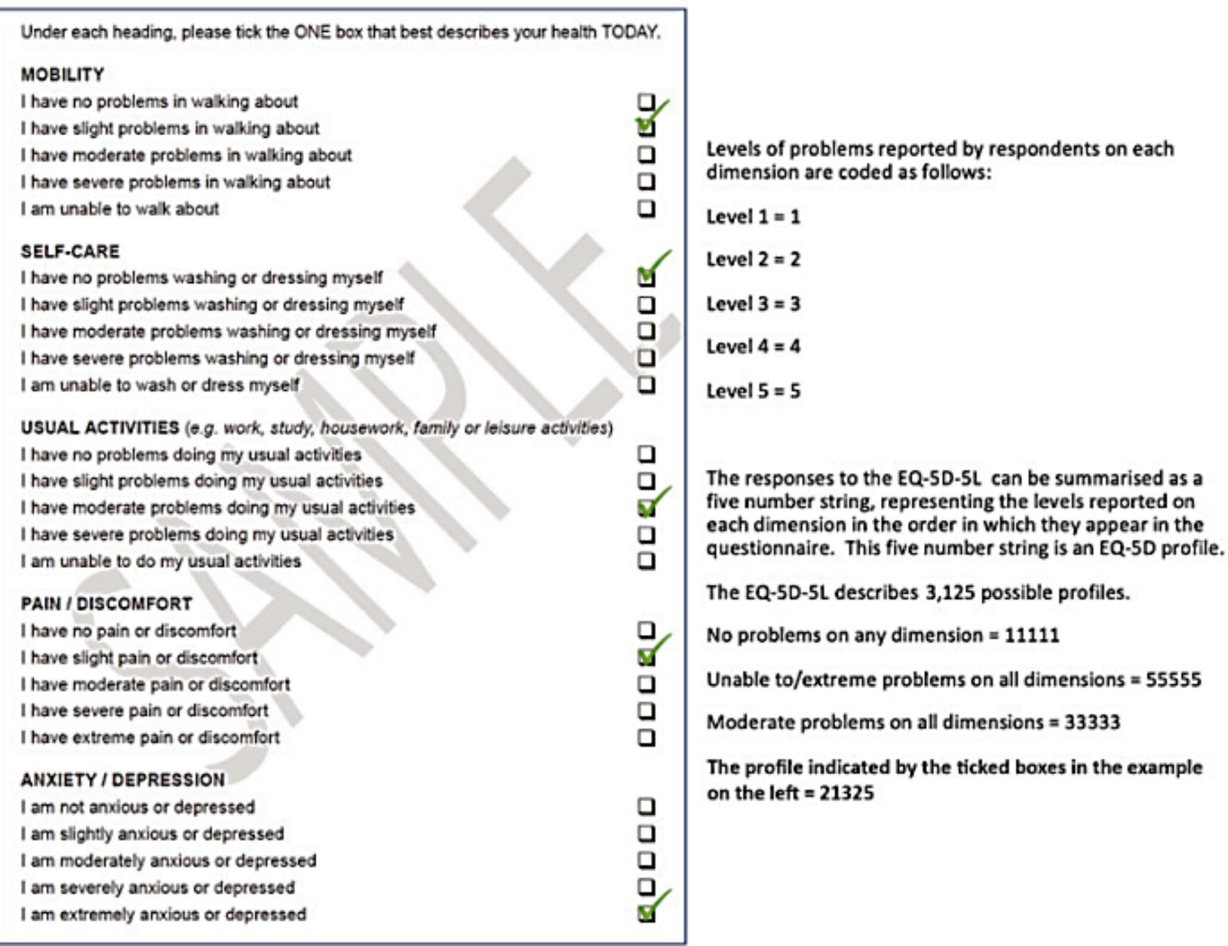
EQ-5D-5 L descriptive system. *Source*: EuroQol Research Foundation. *EQ*-*5D*-*5 L User Guide*, *2019*. Latest version available from: https://euroqol.org/publications/user-guides

The EQ-5D is a concise, generic measure of self-reported health, developed by EuroQol group [22, 23]. Designed as a self-completion questionnaire, it embodies two components, a health state description followed by an evaluation. The ‘5D’ in its name refers to its use of five dimensions to describe health states domains: Mobility (M), Usual Activities (UA), Self-care (SC), Pain & Discomfort (PD) and Anxiety & Depression (AD). A verbal 5-point rating scale that allows the distinction of five levels (‘5 L’) of severity assesses them: Level 1: no problems; Level 2: slight problems; Level 3: moderate problems; Level 4: severe problems; Level 5: extreme problems per dimension and providing a 1-digit number for each dimension. The descriptive system, is shown in Fig. 1. Respondents were asked to tick boxes to indicate the level of problem they were experiencing on each of the five dimensions. The combination of these ticks under each dimension describes that person’s EQ-5D self-reported health state, often called an ‘EQ-5D profile’. Each level of problem experienced is rated from one to 5 points according to self-experienced severity (one – no problem, five - severe problems). Only one case must be completed. The digits for the 5 dimensions health states were directly converted into Romanian-specific single index values (utilities) using country specific value sets [22]. This was the basis for the analysis. The second part of the questionnaire is the EQ VAS, so called because it incorporates a Visual Analogue Scale. This captures the respondent’s overall assessment of their health on a scale from zero (worst health imaginable) to 100 (best health imaginable).

The test was translated in Romanian and applied to 38 patients with abdominal wall hernias for internal validation in 2020. Cronbach’s α was 0.86 suggesting a shared dimensions of the items. For the test – retest reliability, participants completed the questionnaires after 3.9 ± 0.97 h (mean ± SD) apart. The ICC was found to be 0.87 (95% CI: 0.85–0.9). Based on these data we considered that the test could meet our QoL assessment needs with minimal risk of bias.

### Outcomes

The main outcomes of interest were 30-day wound events and general complications including 90 days mortality. Post-operative wound events included Surgical Site Infection (SSI), Surgical Site Occurrence (SSO), and Surgical Site Occurrences Requiring Procedural Intervention (SSOPI). According to the Centre for Disease Control and Prevention (CDC), SSI was defined as superficial, deep or organ space [24]. Surgical Site Occurrence included any SSI, in addition to wound cellulitis, non-healing incisional wound, skin or tissue ischemia, skin or soft tissue necrosis, fascial disruption, serous or purulent wound drainage, stich abscess, Seroma, hematoma, infected or exposed mesh, enterocutaneous fistula. Procedural interventions which were considered SSOPI included wound opening and/or debridement, stich removal, percutaneous drainage, partial and/or complete mesh removal. Length of hospital stay, 30-day readmission and mortality were also analysed. Patients having multiple wound complications such seroma and infection, dehiscence and infection, were classified as the most severe. The follow – up for this study was performed by personal examination of one of the two senior authors. The patients were examined in the outpatient clinic at 1 month, 6 months, 12 months, and yearly or whenever necessary if clinical complaints were described. A recurrence was defined as a bulge at the operative site at any moment of the follow-up; clinical suspicion was confirmed by a native CT abdominal – pelvic CT scan. All postoperative data were collected unblinded.

### Statistical analysis

Data were tabulated as mean ± standard deviation (SD). Continuous variables were analysed by ANOVA variance test followed by unpaired two tails Student’s *t* test assuming unequal variance and the binary outcomes with the Chi-square (χ2) test. Pearson correlation coefficients with the regression equation were used to estimate the correlation between different cofounders. Correlations were considered ‘strong’ if the coefficient value lied between 0.50 and 1, were said to be ‘moderate’ if the value lied between 0.30 and 0.49; and were considered ‘weak’ correlations if the coefficient lied below 0.29. Multivariate logistic regression models were built with all the variables as the outcomes of interest for QoL and AWF, adjusting for identified confounders. In addition to the variables of interest, the following were included for adjustment: age, rank of recurrence, severity of comorbidities score, BMI, length, width and area of the defect, and pain. Multivariate models were calculated with the linear logistic regression and the results were shown with the Odd’s ratio (OR) and the 95% confidence interval (CI). A Receiver Operating Characteristics (ROC) curve was designed to determine the specificity and sensitivity of the cofounders in relation with the total amount of QoL and AWF. Youden index was determined to calculate the optimal cut-off value for each cofounder. Values of the OoL index were associated with the value of the standard error of mean (SEM) [23]. Probabilities smaller than 0.05 were considered as statistically significant. SPSS statistic version 23.0, 2018 (Ch., Ill) was used to perform the statistical analysis.

## Results

### Patient characteristics and clinical outcome

**Fig. 2:**
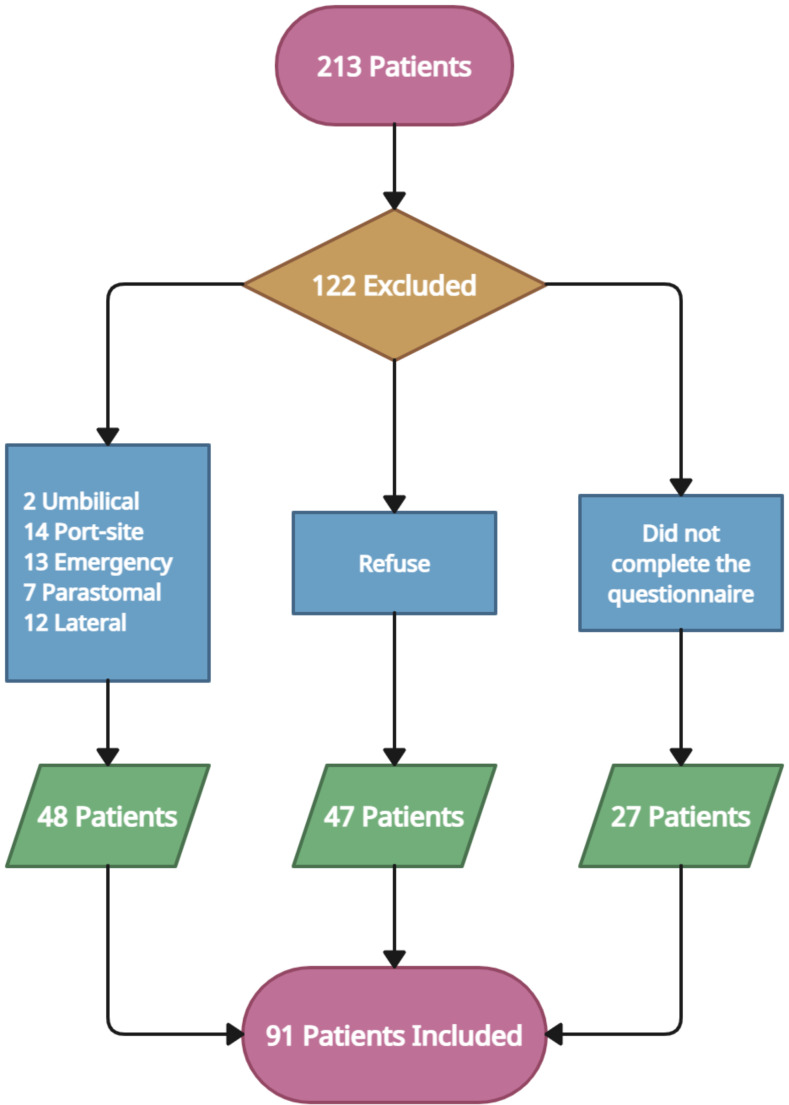
The flow chart of patient’s distribution according to inclusion and exclusion criteria

In the referred interval of time, 213 patients with incisional hernias of various types and locations were admitted and operated in the Department of Surgery. Of these, 91 met the inclusion criteria, agreed to participate, signed the informed consent and were included in the study (Fig. 2). Most of the patients were obese, 62 of them having a BMI over 30 kg/m^2^. Demographic data, comorbidities and preoperative characteristics are represented in Table 2.

**Table 2:** Preoperative demographic characteristics of studied patients. BMI – body Mass Index; COPD – chronic obstructive Pulmonary Disease; CVD – Cardiovascular Disease

Variable	
Age (years) (mean ± SD) (range)	59.42 ± 12.28 (25–80)
Gender (male/female)	35/56
BMI (mean ± SD) (range)	32.02 ± 5.07 (20–47)
Diabetes (%)	27(30%)
Obesity (BMI > 30 kg/m^2^	62 (68%)
COPD (%)	8 (9%)
CVD (%)	56 (62%)
Smokers (active) (%)	29 (32%)
ASA score (%)	ASA 1–7 (8%)
	ASA 2–78 (86%)
	ASA 3–6 (6%)
Recurrence rank	Primary – 21
	First − 27
	Second − 13 Third − 13
	Forth − 12
	Fifth − 5
Length of the defect (cm) (mean ± SD) (range)	12.6 ± 6.88 (3–30)
Width of the defect (cm) (mean ± SD)	10.1 ± 5.5 (2–30)

Intraoperatively, the location and dimensions of the defect were confirmed and compared with the preoperative values; there were no significant differences (data not shown). According to the defect width, Rives–Stoppa repair was performed in 35 patients, Anterior Component Separation with on lay mesh reinforcement in 13 patients, and Posterior Component Separation with Transversus Release in 43 patients. Intraoperative details are depicted in Table 3.

**Table 3:** Intraoperative details: VHWG (Ventral Hernia Working Group), defect measurement, type of surgery, operative time, estimated blood loss, and intraoperative associated injury rate

Variable	Number (%)
Grade 3 VHWG classification	15 (16%)
Width less/equal than 6 cm	25 (27%)
Width between 6 and 10 cm	28 (31%)
Width over 10 cm	38 (42%)
Rives – Stoppa	35 (38%)
Anterior Component Separation (ACS)	13 (14%)
Posterior Component Separation (TAR)	43 (47%)
Operative time (minutes) (mean ± SD)	150.27 ± 52.99 (60–285)
Rives – Stoppa (R-S)	124.84 ± 34.3 (60–210)
Posterior Component Separation (TAR)	174.37 ± 51.31 (80–285)
Anterior Component Separation (ACS)	148.44 ± 39.43 (80–200)
Estimated Blood loss (ml)	134.5 ± 74.94 (0–350)
Bowel injury	9 (10%)
Urinary bladder injury	2 (2%)

Along this period, local and systemic complications were recorded as follow: wound infection – 4 patients (4%), hematoma – 27 (30%), seroma – 26 (29%), skin necrosis – 7 patients (8%), Acute Respiratory Distress Syndrome in 2 patients, and cardiac arrest also in 2. In the ICU were admitted patients with ASA score higher than 3 and with more than two comorbidities. The mean ICU stay was 1.6 ± 1.72 days (1–10 days). Postoperative hospital stay ranged between 4 and 21 days with a mean of 5.52 ± 3.26 days. Global 30 – day mortality was encountered in four patients, and it was due to pulmonary embolism, myocardial infarction, and Clostridial severe infection. Complication rates related to surgical procedure is detailed in Table 4. One-year recurrence was 5% (5 patients).

**Table 4:** Postoperative complication rates related to the procedure

	Wound infection	Hematoma	Seroma	Skin Necrosis	ARDS	Cardiac arrest	Mesh infection	Recurrence
Rives-Stoppa 35 (38%)	0 (0%)	6 (17%)	4 (11%)	0 (0%)	0 (0%)	0 (0%)	0 (0%)	0 (0%)
Anterior Component Separation 13 (15%)	2 (15%)	12 (92%)	13 (100%)	5 (38%)	1 (7%)	1 (8%)	1 (8%)	3 (23%)
Posterior Component Separation 43 (47%)	2 (5%)	9 (21%)	8 (19%)	2 (5%)	1 (2%)	1 (2%)	0 (0%)	2 (5%)
Total: 91	4 (4%)	27 (30%)	26 (29%)	7 (8%)	2 (2%)	2 (2%)	1 (1%)	5 (5%)

### Abdominal wall function and QoL data

At the end of the first year, only 56 patients completely filled out the Quality-of-Life evaluation form.

The mean value of combined score for the preoperative abdominal wall functionality was 4.41 ± 1.67 (2–8) while the mean value of preoperative EQ-5D index was 0.652 ± 0.026 (-0.32–1.00) (see details for each dimension in Table 5). There were no patients with normal abdominal function recorded preoperatively; most of them (48–53%) recorded trace or poor function while only 11 (12%) were with degrees of good functionality. The preoperative QoL was poor and very poor for 48% (44) of the patients who recorded index values less than 0.56 (50% percentile). Only 7% of the patient are recorded with good QoL scores (index values over 0.87–90% percentile). Preoperative EQ-5D index was highly correlated with Combined AWF score (*r* = 0.620; *p* < 0.0001) and the correlation was specific (AUC = 0.799; *p <* 0.0001; asymptotic 95%CI = 0.711–0.923). Univariate analysis shows a significant positive correlation with the age (*p* = 0.004), diabetes (*p* = 0.012), cardiovascular diseases (*p* = 0.016) and smoking (*p* = 0.055). The preoperative QoL index was negatively significantly correlated with the age (*p =* 0.002) but the correlation was not specific except for a Youden index of 62.4 years. Length and width of the defect negatively, significantly, and specifically influenced the value of preoperative index (see Table 6). In multivariate logistic regression, the same factors (except smoking) were identified as independent cofounders directly influencing preoperative QoL (Table 6).

**Table 5:** Postoperative evolution of quality of life dimensions and QoL index EQ-5D. Data are expressed as mean ± SD and mean ± SEM for index values

Dimension /time lapse	Preoperatively	1month postoperatively	6months postoperatively	12months postoperatively
Mobility	3.14 ± 0.99(1–5)	2.08 ± 1.00 (1–4)	1.54 ± 0.72 (1–3)	1.07 ± 0.47 (1–2)
Self-care	3.09 ± 1.1 (1–5)	2.03 ± 1.03 (1–4)	1.48 ± 0.72 (1–3)	1.01 ± 0.41(1–2)
Usual activities	2.98 ± 1.1 (1–5)	1.9 ± 1.00 (1–4)	1.39 ± 0.67 (1–3)	1.01 ± 0.41 (1–2)
Pain/discomfort	2.15 ± 1.01(1–5)	1.53 ± 0.82 (1–3)	1.13 ± 0.48 (1–2)	0.94 ± 0.30(1–2)
Anxiety/depression	1.98 ± 1.11(1–5)	1.49 ± 0.99 (1–5)	1.30 ± 0.86 (1–5)	1.01 ± 0.51 (1–4)
EQ-5D QoL index	0.6521 ± 0.24	0.7861 ± 0.18	0.894 ± 0.09	0.9795 ± 0.05

**Table 6:** Univariate and multivariate analysis related to demographics, comorbidities, defect size, recurrence rate, abdominal wall function, and pain. COPD – chronic obstructive Pulmonary Disease; CVD – Cardiovascular diseases; AWF – abdominal wall function; OR –Odds ratio; 95%CI – confidence interval; AUC – area under curve (was calculated only when the correlation was statistically significant *p* < 0.05)

Variable	Univariate analysis	Logistic regression		
		OR	95% CI	*p*
**Age**	***r*** = -0.324; ***p =*** **0.002; AUC = 0.422**; ***p*** **= 0.649**	0.0681	0.028 − 0.62	**0.038**
Gender	*r* = -0.138; *p* = 0.278	0.71	0.98–1.78	0.22
**Diabetes**	***r*** = 0.261; *p* = 0.012; AUC = 0.659; *p* = 0.026	0.059	0.092–23.45	**0.003**
Obesity	*r* = -0.093; *p* = 0.382	1.501	0.677–3.330	0.318
COPD	*r* = -0.006; *p* = 0.956	1.196	0.995–1.437	0.066
**CVD**	***r*** = 0.325; *p* = 0.002; AUC = 0.573; *p* = 0.013	0.21	0.049–1.09	**0.05**
Smoking	*r* = -0.273; *p* = 0.055	0.40	0.074–2.14	0.28
**Length**	***r*** = -0.385; *p* < 0.0001; AUC = 0.727; *p* = 0.035	0.071	0.07–0.85	**0.027**
**Width**	***r*** = − 0.293; *p* = 0.005; AUC = 0.767; *p* = 0.0117	0.85	0.017–0.479	**0.021**
Recurrent	*r* = 0.200; *p* = 0.168	0.17	0.02–1.54	0.11
**Preoperative AWF**	***r*** = -0.620; *p* < 0.0001; AUC = 0.799; *p* < 0.0001	0.03	0.004–0.26	**0.0012**
**Preoperative pain**	***r*** = 0.442; *p* < 0.0001; AUC = 0.772; *p* < 0.0001	0.029	0.003–0.19	**0.009**

At 1 month postoperatively AWF significantly increased compared to the preoperative values (preoperative total score 4.41 ± 1.67 versus 1-month total score 5.62 ± 2.04; *p* < 0.0001. The number of patients with fair, good, and normal AWF increased from 43 preoperatively to 68 in the first postoperative month (χ^2^ = 8.36; *p =* 0.003). The index score of QoL was generally rated as good and increased to 0.786 ± 0.02 (*p* < 0.0001, 95% CI of difference = -4.35 - -2.88) compared with the preoperative value. Comparing with the preoperative values the difference was statistically significant (χ^2^ with Yates correction of continuity = 15.89; *p =* 0.00067).

**Fig. 3:**
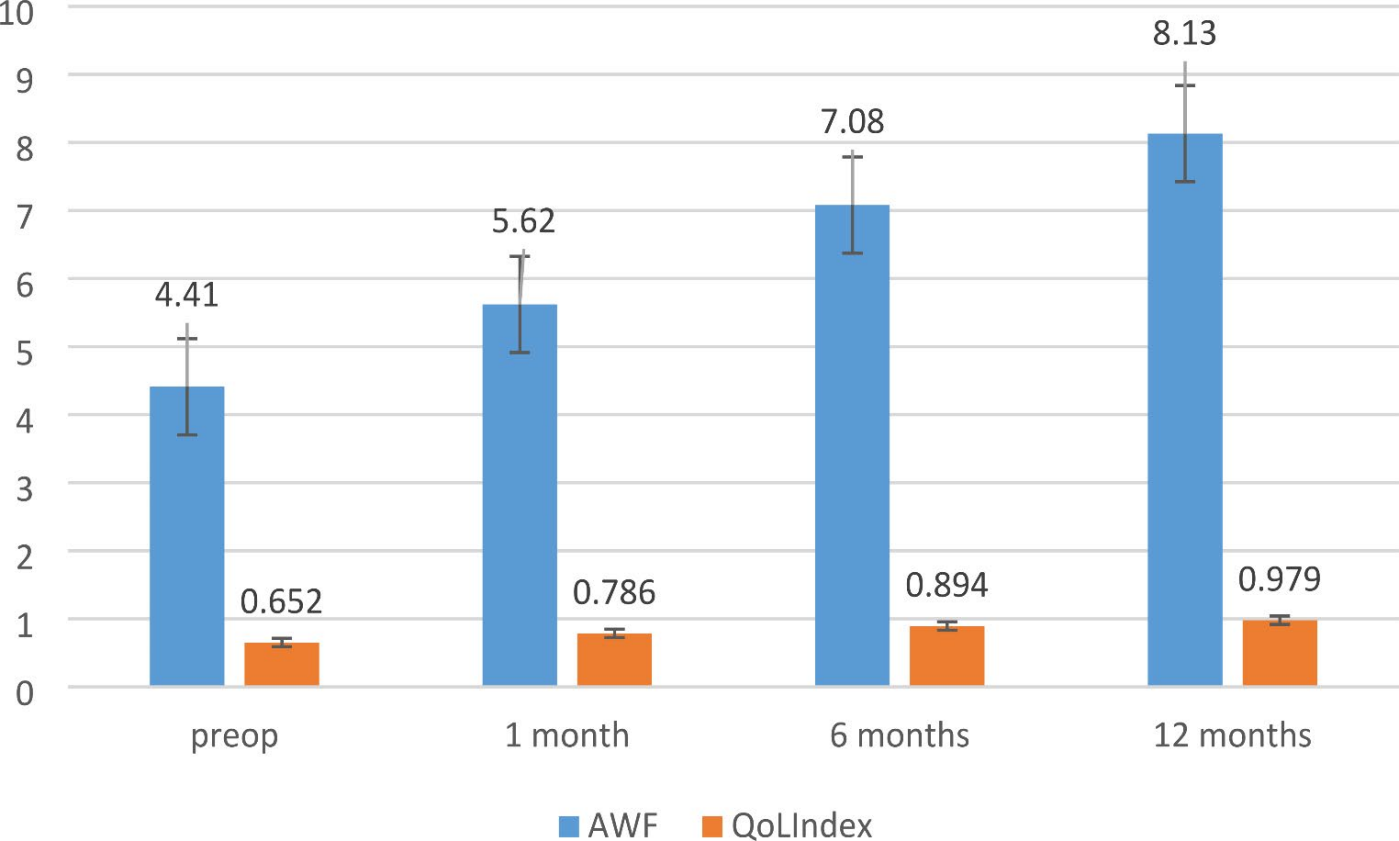
The evolution of abdominal wall functionality in relation to the EQ-5D QoL index. The difference with the 1-month score was significant (*p* < 0.0001; 95% CI of difference = 2.51–3.51). Data expressed as mean ± SD for AWF and as mean ± SEM for QoL index

Abdominal wall functionality increased at 6 months postoperatively comparing to 1-month value of total score (7.08 ± 1.99 *versus* 5.62 ± 2.04) (*p* < 0.01). Of 65 responders, 50 were with good and normal AWF comparing to 31 at 1 month postoperatively (χ^2^ = 22.048 with Yeats correction of continuity; *p <* 0.00001). The mean QoL index score at 6 months postoperatively increased to 0.894 ± 0.011 (range 0.56–1) (*p* = 0.037) (Fig. 3).

At 12 months, only 56 patients completed the evaluation of AWF and only 48 filled the QoL questionnaire. The AWF score increased to 8.13 ± 2.58 (1–10) and the QoL total score to 0.979 ± 0.007 (0.71–1) (Table 5). Good and very good total scores for QoL were recorded for 47 patients (84%) compared to 33 (36%) in the preoperative evaluation (χ^2^ with Yates continuity correction for two degrees of liberty = 46.04; *p* < 0.00001).

In the first postoperative month, the mean percent of increase in the quality of life was 42.34 ± 15.93 (range − 16.3 − 527%) with 11 patients in the 75% percentile with increases over 30.9%. A significant increase with mean percent of 88.89 ± 23.78 (9.89 − 770%) was achieved after the first postoperative year with 34 patients in the 50% percentile (increases over 35.13%). The most significant increase in QoL was achieved after the first postoperative month (*p* < 0.0001, 95% CI of difference − 65.15 - -27.95, t = -5.03), and a smaller insignificantly one for the rest of the period.

Confounders influencing QoL in postoperative period were analysed at 1 and 12 months. At one – month postoperatively QoL was correlated with gender (*p* = 0.02 for females), presence of diabetes (*p =* 0.036), with the length of the defect (*p =* 0.05), presence of wound hematoma (*p =* 0.05), ICU stay (*p =* 0.03), acute respiratory failure (*p <* 0.0001), and preoperative and 1-month total score of AWF (for both *p* < 0.0001). Multilogistic regression associated diabetes (OR = 0.003, 95% CI = 0.004–0.26; *p* = 0.0012), length of the defect (OR = 0.029; 95% CI = 0.003–0.19; *p* = 0.009), wound hematoma (OR = 0.0821; 95% CI = 0.017–0.53; *p* = 0.0022), and intensive care unit stay (OR = 0.21; 95%CI = 0.044–1.07; *p* = 0.041) as the only independent predictors for QoL at 1 month postoperatively. No correlates and predictors for QoL were identified during the 12-month evaluation.

According to the surgical procedure, the only difference of QoL between groups was determined by the dimension of the defect, significantly higher for patients with ACS and TAR (Table 2). The preoperative value of QoL index was significantly lower for patients with TAR compared with Rives – Stoppa (*p =* 0.003; 95% CI of difference 0.073–0.33). There was no difference between the values in all moments of evaluation despite surgical procedure.

**Fig. 4:**
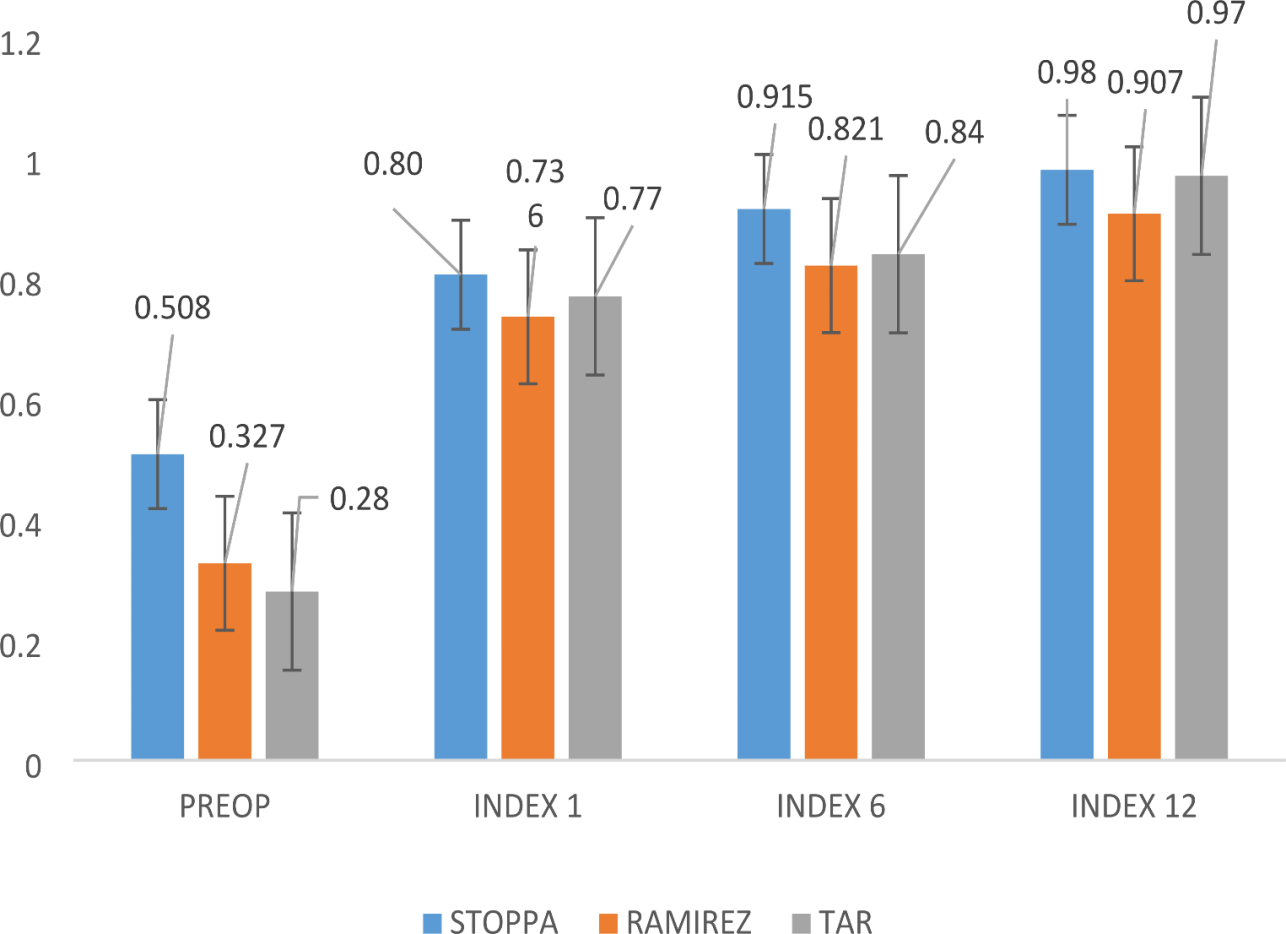
EQ-5D QoL index in relation to type of surgery; Preop – preoperatively; Index 1–1-month index value; Index 6–6 months index value; Index 12–12 months index value. Preoperatively scores for the QoL were lower for the patients with Rives – Stoppa repair comparing with ACS (*p* = 0.007) and TAR (*p =* 0.00023). Quality of life was insignificantly different in patients with ACS compared to TAR patients (*p =* 0.528). Data expressed as mean ± SEM

The mean values of the QoL scores according to the surgical procedure are represented in Fig. 4. Preoperatively, we recorded almost equal mean scores for the QoL, but they were lower for the patients with Rives – Stoppa repair and the difference was significant comparing with ACS (*p* = 0.007) and TAR (*p =* 0.00023). Quality of life was insignificantly different in patients with ACS compared to TAR patients (*p =* 0.528). Postoperatively, for all evaluation intervals, the QoL for patients with underlay repair was significantly increased compared to patients with anterior component separation (Fig. 3).

## Discussion

Traditionally the success of a surgical procedure was evaluated by measuring short –term outcomes as utilization measures (length of hospital stay, duration of surgery, readmission, and post procedural complications). However, they do not necessarily capture the effects of health outcomes with significant importance [25].

Abdominal wall hernia patients have different experience both pre and postoperatively compared to the daily lives of the “normal population” and have often suffered a significant surgical history with subsequent physical and psychological impact [26, 27]. This is the reason why the effects of surgery on health-related quality of life are arguably the most important outcome to measure in evaluating the effectiveness of surgical care. Incorporation of the patient perspective on health and QoL produced in last decade a growing body of literature [28].

Our results reflect the natural progression of postoperative recovery. Baseline patient characteristics showed significant differences between preoperative and postoperative QoL index value along studied intervals of time. Our patients with symptomatic IH were more likely to be female and more likely to have an increased BMI; the results are in accordance with Schlosser et al. [29]. In patients with small defects with high preoperative indexes of QoL, the postoperative QoL records only a small increase. This could be a debatable topic related to the moment for surgery and could consider watch full waiting as an option in patients with modifiable risk factors until the correction. We could not find in the literature such relation.

Our study shows a constant positive impact on QoL despite surgical repair, that is significantly improved when compared to preoperative values for all three studied surgical techniques. Even if the increase was not significant for all patients the rate of improved QoL at the end of the first year was superior to Langbach et al. who reported an overall improved status in only 63%, with SF 36 questionnaire [30]. According to the same authors, an unmodified status was recorded in 20% and a worst one in 17%. A corresponding fraction of satisfied patients was reported in other studies [31, 32]. It is difficult to explain such differences through the terms of hernia characteristics, surgical procedure, or the number of comorbidities because these data are equal. More likely, it is a matter of self-reporting in accordance with cultural differences but apart from these differences, there is a broad array of factors difficult to define. One of them is probably the relation between expectations and experience. Although successful repair was achieved in 84% of the patients, an important ratio report dissatisfaction with physical function, aesthetic results and psychic condition. The results are in accordance with van Veenendaal et al. who reports 17% of patients with a worse status compared to before operation [33].

Initially, the mobility function remains quite low with a mean overall increase slightly over 40% but this increase is highly correlated and parallel with the increase of abdominal wall functionality. This, in correlation with the increase of “self-care “and “activity” domains, suggest that the physical component of the QoL is almost completely recovered with more than 80% after the first postoperative year despite the presence of complications. The lower scores on physical domain generally seen among women [29, 34] were not confirmed in our study. The worst improvement was recorded for the “mental” domain and was dominated by the fear of recurrence, mesh related complications and subsequent surgical intervention (data not shown).

Objective measurements of the abdominal wall functionality are difficult to be obtained for the current practice because of the major barriers in choosing the optimal method for its representation. Dynamometry has been established as a reliable and effective method to evaluate AWF both with static and dynamic work [35, 36]. Despite offering an objective approach with less subjective interpretations bias, the method is highly expensive, has limited flexibility for conducting tests in other locations, and the number of investigated patients is too small to have sound conclusions [37–39]. Our study, using clinically validated tests [40], demonstrates a profound alteration of the abdominal wall musculature, especially in patients with large incisional hernias and this limitation was highly correlated with the preoperative QoL in the developed regression model. These results are in accordance with the study of Strigård et al. who demonstrated with dynamometric evaluation a decrease of functionality in patients with hernias larger than 10 cm wide [37].

As the area of the defect increases, there is a proportional decrease in the AWF and also in QoL; patients with defects ranging from 6 to 10 cm width have altered but better AWF comparing to patients with defects larger than 10 cm and the difference was significant. There is such a strong correlation that it seems safe to conclude that the width of the defect is the one of the most important determinants for the degree of lost function [21].

Abdominal wall specific surveys have been developed to assess AW – QoL with greater utility than general health surveys [10]. So, why we choose a non-specific questionnaire? The EQ-5D was suitable and adaptable for translation and implementation to Romanian population. In addition, the value sets for Romanian population were available [23] and allow us to easily calculate index QoL. The EQ-5D tailored for the pre and postoperative states provides 5 items quickly and easily completed by the patient. Our preoperative study revealed a good acceptability, reliability, and validity. The design of the questionnaire quantifies generic patient reported outcomes based on all affected incisional hernia domains (mobility, self-care, usual – activities, pain, and anxiety/depression). Even if the response rate after 1 year was only 56%, there are studies that quote response rates in the low 30% range as adequate, with a mean response rate of 50–60% [1]. Results from each symptom can be aggregated to calculate an overall index limitation. Overall, our questionnaire demonstrates a large effect size between pre and postoperative scores and captures the improvement in QoL. QoL scores from EQ-5D have been shown to be comparable to those of Carolinas Confort Scale [8].

Our study is not without limitations. Although data was collected in a prospective manner, this study remains observational and current data should cautiously interpret. Our data cannot be extrapolated to another population undergoing surgical repair because they are specific to a subset with cultural, ethnic, and educational particularities. Being a single experience team cohort of patients’ needs validation in other Romanian population. A team of dedicated surgeons performed all surgical procedures, can represent a selection bias and the results cannot be extrapolated to general surgical population. The follow-up is relatively short (many of the sequels which can severely impact QoL are reported later) and the small sample size with reduced rates of responses can be considered a selection and collection bias. Last, our cohort consists only of elective patients and this can be considered another selection bias.

## Conclusion

A significant overall improvement in all five components of QoL measured with the help of EQ 5D questionnaire was demonstrated.

This improvement is dependent by hernia size, and some individual patient’s factors (diabetes, cardiovascular diseases, and age over 60 years).

Further studies should delineate what factors affect EQ. 5D scores and to validate the questionnaire to a population level as being an effective tool for hernia assessment.

## Electronic supplementary material

Below is the link to the electronic supplementary material.

Supplementary Material 1

Supplementary Material 2

Supplementary Material 3

Supplementary Material 4

Supplementary Material 5

Supplementary Material 6

Supplementary Material 7

Supplementary Material 8

Supplementary Material 9
